# OsCBL1 modulates rice nitrogen use efficiency via negative regulation of *OsNRT2.2* by OsCCA1

**DOI:** 10.1186/s12870-023-04520-4

**Published:** 2023-10-18

**Authors:** Zhao Hu, Yutan Guo, Suping Ying, Yunting Tang, Jiawei Niu, Ting Wang, Ruifeng Huang, Hongwei Xie, Wenya Wang, Xiaojue Peng

**Affiliations:** 1 Key Laboratory of Molecular Biology and Gene Engineering of Jiangxi Province, College of Life Science, Nanchang University, Nanchang, 330031 China; 2 Department of Chemistry, University of Kentucky, Lexington, KY USA; 3 Jiangxi Super-rice Research and Development center, National Engineering Laboratory for Rice, Nanchang, China; 4 Msc Applied Genomics, Imperial College London, London, UK

**Keywords:** *Oryza stativa*, *OsCBL1*, *OsCCA1*, *OsNRT2.2*, NUE

## Abstract

**Background:**

For cereal crop breeding, it is meaningful to improve utilization efficiency (NUE) under low nitrogen (LN) levels while maintaining crop yield. *OsCBL1*-knockdown (*OsCBL1-*KD) plants exhibited increased nitrogen accumulation and NUE in the field of low N level.

**Results:**

*OsCBL1*-knockdown (*OsCBL1-*KD) in rice increased the expression of a nitrate transporter gene *OsNRT2.2*. In addition, the expression of *OsNRT2.2*, was suppressed by OsCCA1, a negative regulator, which could directly bind to the MYB-binding elements (EE) in the region of *OsNRT2.2* promoter. The *OsCCA1* expression was found to be down-regulated in *OsCBL1-*KD plants. At the low Nitrogen (N) level field, the *OsCBL1-KD* plants exhibited a substantial accumulation of content and higher NUE, and their actual biomass remained approximately as the same as that of the wild type.

**Conclusion:**

These results indicated that down-regulation of *OsCBL1* expression could upregulate the expression of *OsNRT2.2* by suppressing the expression of *OsCCA1*and then increasing the NUE of *OsCBL1-KD* plants under low nitrogen availability.

**Supplementary Information:**

The online version contains supplementary material available at 10.1186/s12870-023-04520-4.

## Introduction

Nitrogen (N) is one of the essential fertilizers that farmers utilize in their fields since insufficient N availability of N in fields can severely restrict inhibit plant growth and resulted in losses in crop productivity [1]. However, excessive N fertilizers has become a global problem that has seriously harmed the ecosystem and biodiversity, including by raising greenhouse gas levels and accelerating water eutrophication [2]. Improving nitrogen use efficiency (NUE) in crops has emerged as one of the most practical strategies to reduce or replace fertilizer overcome in crop production and preventing environmental degradation. This is done to eliminate the pollution from N as field fertilizer and maintain nutrient homeostasis.

Nitrate ( NO_3_^−^), is one of the main sources of nitrogen, which accounts for 40% of the total nitrogen resource for rice through the nitrification in the rhizosphere [3]. Depending on the environmental availability of NO_3_^−^, the plant may utilize NO_3_^−^ through two different uptake systems: high- (HATS) and low (LATS) -affinity transport systems, which adapt plants to low or high NO_3_^−^ concentrations in soil [4]. In recent years, related to these two NO_3_^−^ -uptake systems, several nitrogen assimilation- and transport-related genes have been identified that could boost the NUE in rice. Increasing expression of a high-affinity NO_3_^−^ transport protein, OsNRT2.1/2.2, can improve NUE and yield in rice, according to research by Chen’s group [5]. Meanwhile, Fan’s group reported that increasing expression of *OsNRT2.3b*, another high-affinity NO_3_^−^ transport gene, could also enhance NUE and rice grain yield in the field [6]. Moreover, over-expression of *OsNRT1.1 A* and *OsNRT1.1B* (two low-affinity NO_3_^−^ transporter genes in rice) could increase the NUE of rice by regulating nitrate uptake [7, 8]. In addition, lots of nitrogen assimilation- and transport-related genes (OsNR2, OsNIR1, OsGS1:1, OsGOGAT1, OsAMT1;1, OsAMT1;3), and transcription regulatory factors (OsNLP3, OsNLP4, OsGRF4 and OsNGR5) have been shown to be involved in improving rice NUE [9–18].

Recently, a circadian rhythm factor, OsCCA1, for photoperiodic flowering has been found associated with NUE of rice [19, 20]. Zhang et al. reported that OsCCA1/OsNhd1 concomitantly mediates flowering time and impacts NUE by regulating OsHd3a and several genes related to N assimilation and amino acid transport [21]. Li et al. following showed that Nhd1, a rice circadian clock regulator, can directly activate the expression of a high-affinity ammonium transporter gene *OsAMT1;3* and a dual-affinity nitrate transporter gene *OsNRT2.4*.They also found that mutations of *nhd1* increased N accumulation and NUE in plants in low N supply paddy fields [22].

Nitrate was also reported to play an important role as a signaling molecule to regulate plant growth and gene expression [23, 24]. The nitrate transporter NRT1.1/CHL1, which also acts as a nitrate sensor, could detect and initiate responses to different levels of nitrate in the environment and regulate gene expression in cells [24]. Calcium is known as a second messenger in the plant signal transduction pathway. Riveras et al. reported that nitrate treatment could raise the cytoplasmic Ca^2+^ concentration, and the fluctuations in intracellular Ca^2+^ concentration could influence the expression of nitrate-responsive genes [25]. In *Arabidopsis*, Liu et al. revealed a new function of Ca^2+^-sensor protein kinases (CPKs), as a master regulator to orchestrate nitrate-activated signal [26]. The nitrate transceptor NRT1.1 and a cyclic nucleotide-gate channel (CNGC15) could constitute a nitrate-sensing switch and generate the nitrate-induced Ca^2+^ influx into the cell [27]. These reports suggested a close association between the nutrient-sensing mechanism for nitrate and cellular calcium signals, however, the mechanism of how nitrate-induced calcium fluctuations are decoded and transmitted in the cell remains unknown.

Yang et al. found that OsCBL1, a calcium sensor, is involved in both nitrate signaling and the regulation of rice seedling growth [28]. Previously, our study revealed that in the *OsCBL1*-KD plants, the inhibition of rice seedling growth does not depend on the deficiency of nitrogen in vivo, but rather on the availability of nitrogen present in the surrounding environment [29]. In this study, we observed that *OsCBL1-*KD plant can accumulate more nitrate from nutrient solution and exhibited elevated expression of a nitrate transporter gene *OsNRT2.2.* The increased expression of *OsNRT2.2* was prompted by the downregulating of a negative transcription factor, *OsCCA1*, in *OsCBL1-*KD plants. For *OsCBL1-*KD rice grown under low N levels in the field, we observed a remarkable increase in both nitrogen content and NUE. Our findings reveal an interplay between cellular nitrate response and calcium-related signal pathways in rice, providing a promising novel strategy to improve NUE for plants under low nitrogen conditions.

## Results

### The growth inhibition caused by*OsCBL1*-KD is associated with nitrate

**Fig. 1:**
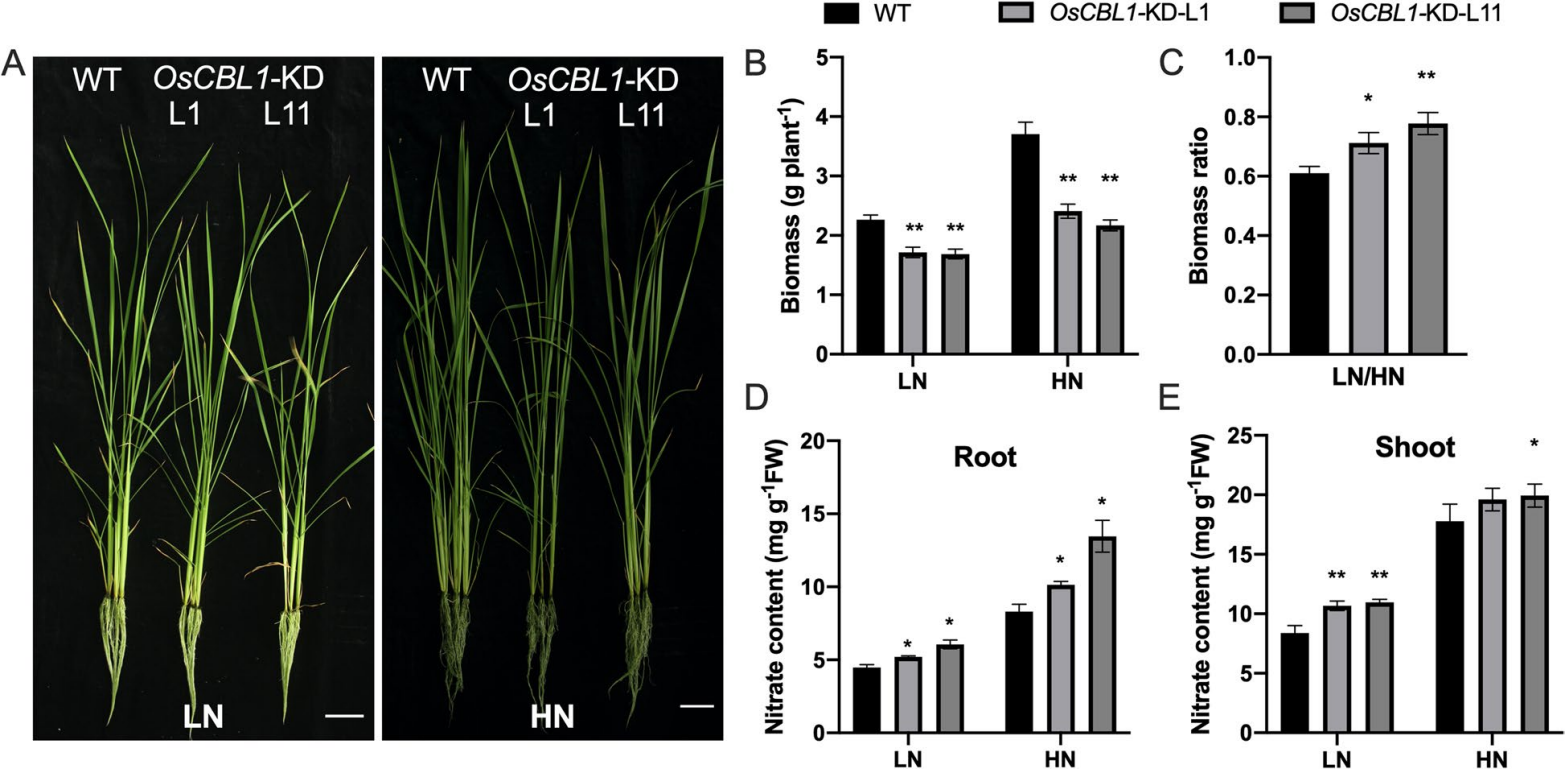
OsCBL1 modulates rice seedling growth associated with nitrogen. **A** Growth phenotype of WT and *OsCBL1*-KD plants at 30 days under high N (5 mM KNO_3_) and low N (0.2 mM KNO_3_) conditions. Scale bars, 5 cm. Each image is representative of triplicates for WT, L1 and L11, a total of 9 rice plants. **B** The biomass of WT and *OsCBL1*-KD plants in HN and LN conditions. *n* = 9 biologically independent samples. The error bars represent ± SDs. **p < 0.01 compared to the WT (t-test). **C **The biomass ratio assays of WT and *OsCBL1*-KD plants from LN to HN conditions. *n* = 9 biologically independent samples. The error bars represent ± SDs. *p < 0.05, and **p < 0.01 compared to the WT (t-test). **D**, **E** Nitrate content in root and shoot of WT and *OsCBL1*-KD plants under HN and LN conditions. *n* = 3 biologically independent samples. The error bars represent ± SDs. *p < 0.05, and **p < 0.01 compared to the WT (t-test)

Our previous study in rice demonstrated that nitrate can help to partially recover the biomass lost as a result of *OsCBL1* knockdown [29]. To further investigate the relationship between OsCBL1 and nitrate, we cultivated rice plants of *OsCBL1*-KD and WT under High N (5 mM KNO_3_) and Low N (0.2 mM KNO_3_) using the Kimura B nutrient solution. We analyzed their biomass, as described in Materials and Methods 2.1. WT plants displayed higher biomass accumulation than *OsCBL1*-KD plants in both HN and LN conditions (Fig. 1B). However, for *OsCBL1*-KD plants, the relative loss of plant biomass between HN and LN conditions is much less than for WT plants. When transitioning from HN to LN nutrient solution, the biomass of WT plants decreased by 39%, whereas *OsCBL1*-KD plants only decreased by 23-29%, (Fig. 1C). The biomass ratio (LN/HN) of *OsCBL1*-KD plants (0.71–0.77) is significantly higher than WT plants (0.61) (Fig. 1C). Considering growing organs at seedling stage, the nitrate content of root s and shoots was determined individually for both WT and *OsCBL1*-KD plants. Compared with WT, *OsCBL1-KD* plants actively accumulated much higher nitrate content in both roots and shoots under HN and LN conditions (Fig. 1D and E). These results showed that the knockdown of *OsCBL1* could increase nitrate content and improve the LN tolerance for rice seedling. These findings collectively suggested that the nitrate is related to the loss of biomass in rice seedling with *OsCBL1* knockdown under hydroponic conditions.

### *OsCBL1* negatively regulates the expression of *OsNRT2.2*

**Fig. 2:**
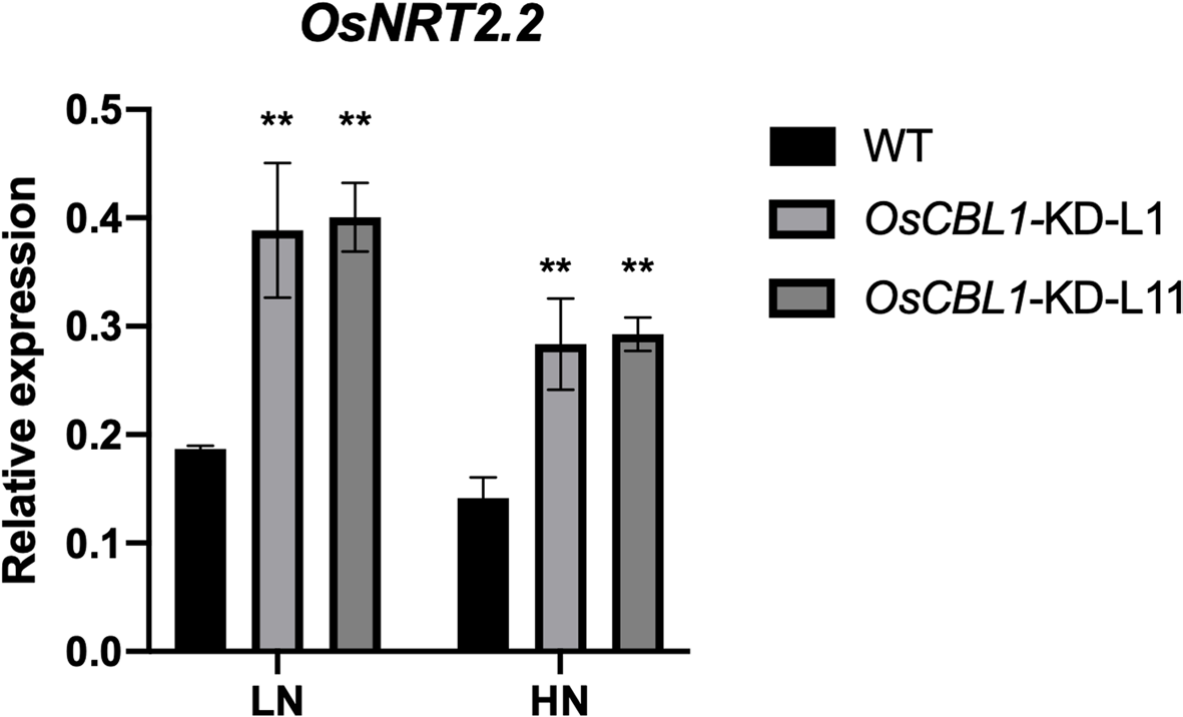
The expression of *OsNRT2.2* in WT and *OsCBL1*-KD plants. *n* = 3 biologically independent samples. The error bars represent ± SDs. **p < 0.01 compared to the WT (t-test)

Since *OsCBL1* modulated nitrate content and N-promoted biomass accumulation in rice (Fig. 1), we investigated whether this observation could be related to some nitrate transporters. We selected five genes from the NRT2 family (*OsNRT2.1*, *OsNRT2.2*, *OsNRT2.3*, *OsNRT2.4*, *OsNRT2.5*) and five from the NRT1 family (*OsNRT1.1 A*, *OsNRT1.2*, *OsNRT1.4*, *OsNRT1.5 A*, *OsNRT1.7*) and compared their transcript levels in both WT and *OsCBL1*-KD plants. Under both HN and LN hydroponic conditions, only the NRT2 family gene *OsNRT2.2* was significantly up-regulated in *OsCBL1*-KD plants, in comparison with WT (Fig. 2, Fig. S1). No other changes were observed in the transcripts level of *OsNRT2.5, OsNRT2.3, OsNRT1.2*, *OsNRT1.4*, *OsNRT1.5 A*, and *OsNRT1.7* (Fig. S1). Additionally, in *OsCBL1-KD* plants, the expression of *OsNRT2.1, OsNRT1.1 A* and *OsNRT2.4* was stimulated only under the LN condition, but not under HN condition (Fig. S1).

Previous research has shown that overexpression of *OsNRT2.1/2.2* promotes the accumulation of nitrate and increases biomass production in rice compared to WT plants [30]. Thus, we infer that the modulations of nitrate content and the promotion of N-induced biomass accumulation by *OsCBL1* could be explained by the elevated expression of *OsNRT2.2*.

### OsCCA1 is a negative regulator of*OsNRT2.2*

To understand the mechanism promoting the expression of *OsNRT2.2* in *OsCBL1-KD* plants, we tried to identify the transcription factor that regulates the expression of *OsNRT2.2.* We conducted a Yeast One-Hybrid library screening assay by employing 1.5 Kb bait sequence (*P1*) from the *OsNRT2.2* promoter to screen the libraries. Several clones were selected from the final dual selective medium plate, and the sequencing results are shown in Table S1. Among those candidate clones, OsCCA1/OsNhd1, a transcription factor from the MYB (myeloblastosis) family (Fig. S2) [31], attracted the most of our interest among those candidate clones. Thus, *OsCCA1*, a circadian clock gene in plants, was chosen as a candidate gene for further studies.

**Fig. 3:**
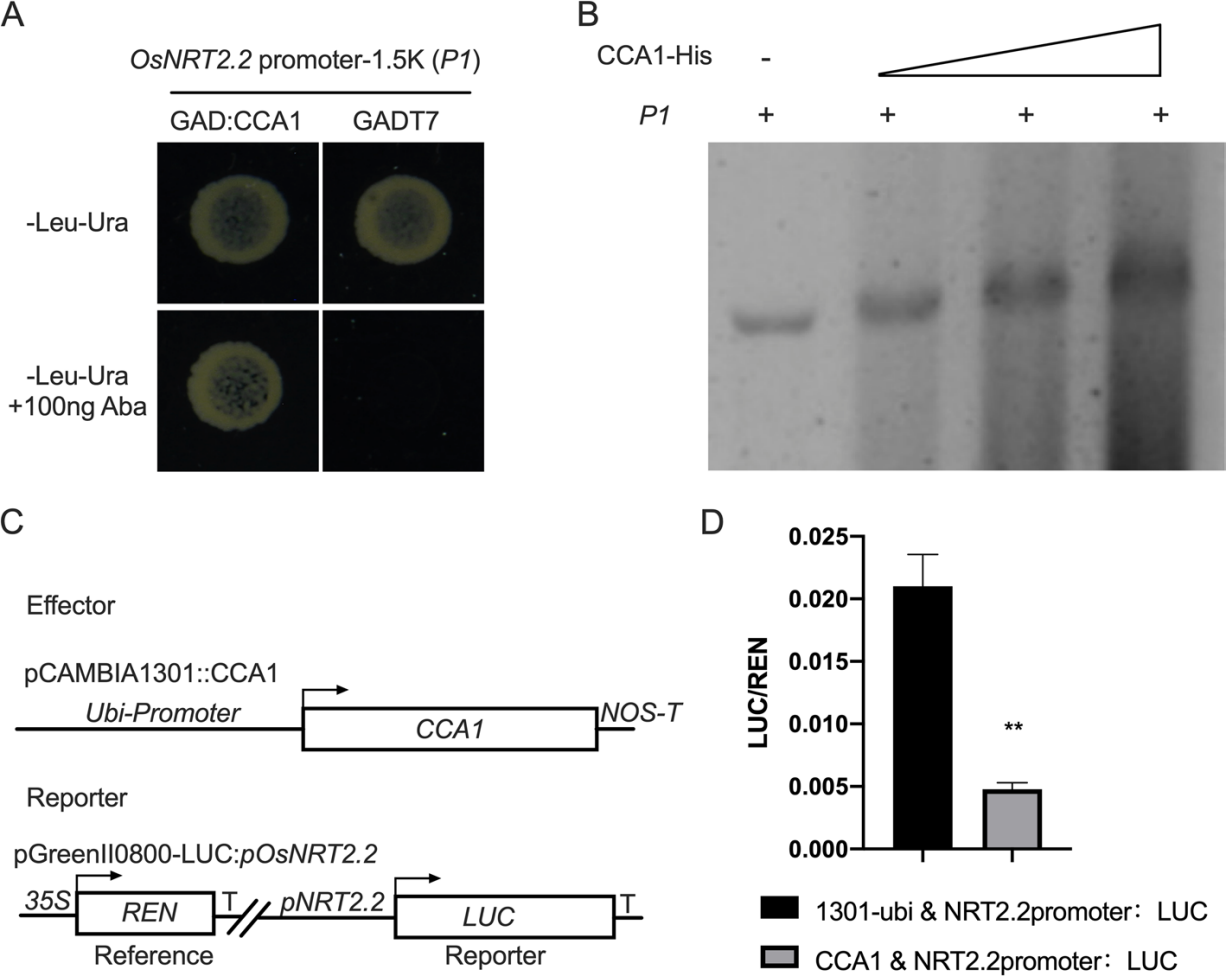
OsCCA1 can directly inhibit the expression of *OsNRT2.2*. **A** Yeast one-hybrid analysis of OsCCA1 bind to the *OsNRT2.2* promoter. **B** DNA binding activities of OsCCA1 to *OsNRT2.2* promoter was tested by EMSA. **C** Effector, reporter, and reference constructs were used. Arrowheads indicate transcription start sites, and NOS-T represents a polyadenylation signal from the nopaline synthase gene. **D** Dual-luciferase reporter analyzes the transcriptional regulation of *OsNRT2.2* by OsCCA1 in rice protoplasts. *n* = 3 biologically independent samples. The error bars represent ± SDs. **p < 0.01 compared to the empty vector (t-test)

To obtain more evidence for the physical interaction between OsCCA1 and *OsNRT2.2* promoter, two constructs (pAbai*-P1* and pGADT7*-OsCCA1*) were created and transformed into the Y1H Gold yeast strain. The interaction between OsCCA1 and *OsNRT2.2* promoter was confirmed as the transformant colonies grew on a medium containing SD/-Leu/-Ura/+Aba (100 ng) (Fig. 3A). An electrophoretic mobility shift assay (EMSA) was also performed to verify this interaction in vitro. Full-length *OsCCA1* fused with a His-tag was expressed in *E. coli* and purified. In the EMSA assay, a significant mobility shift was observed when the *P1* probe was incubated with the His-OsCCA1, indicating an interaction between the OsCCA1 and *OsNRT2.2* promoter (Fig. 3B, Fig. S3). To determine whether OsCCA1 was a negative or positive regulator of *OsNRT2.2*, a reporter gene *LUC* under the control of the *OsNRT2.2* promoter was co-transfected into protoplasts alongside an effector plasmid for expression of OsCCA1 (Fig. 3C and D). As shown in Fig. 3D, the activity of *OsNRT2.2* promoter was inhibited when OsCCA1 was co-expressed. Collectively, these results demonstrated that OsCCA1 exerted a negative effect on the *OsNRT2.2* promoter and directly repressed the expression of *OsNRT2.2*.

### OsCCA1 can bind directly to the MYB-binding elements on the *OsNRT2.2* promoter

**Fig. 4:**
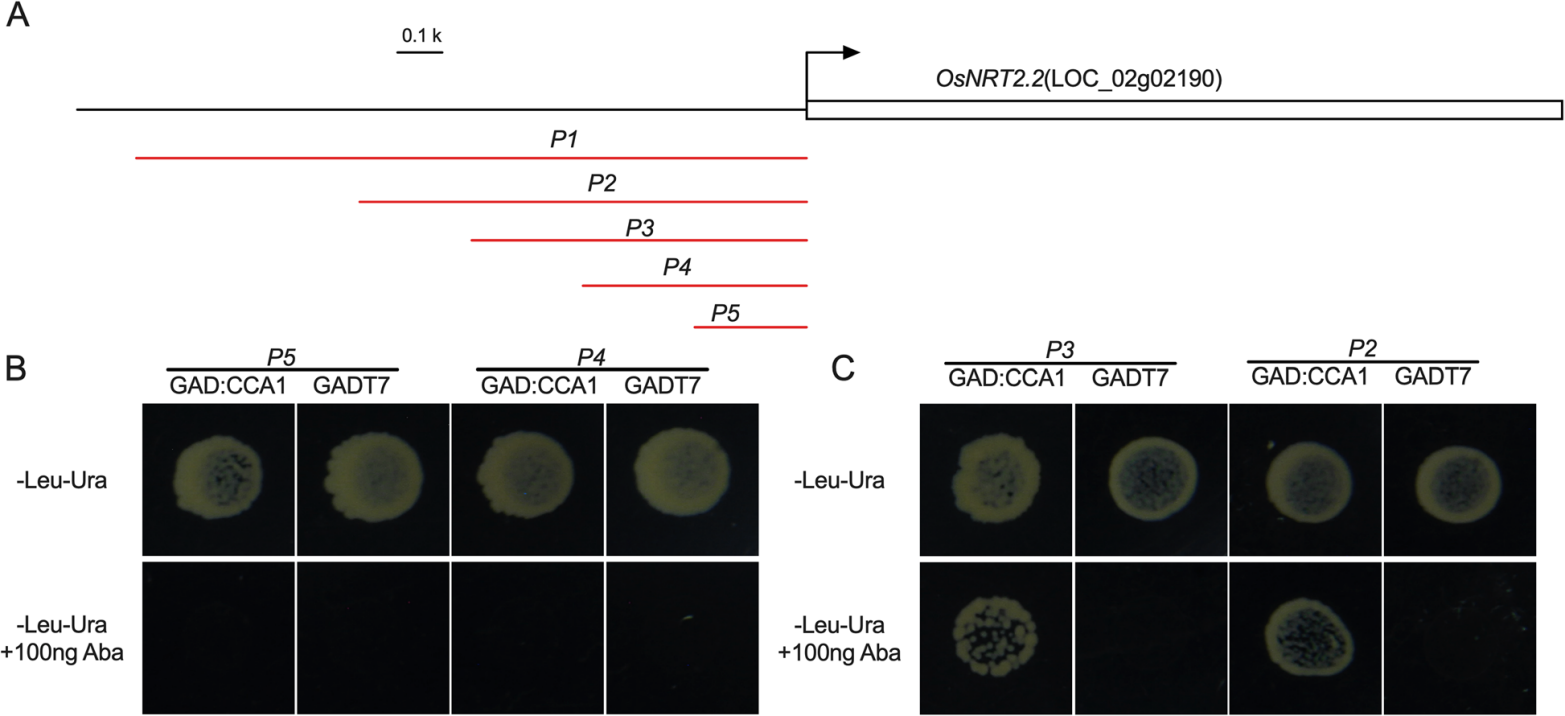
Yeast one hybrid system assay the binding activity of OsCCA1 to *P2*, *P3*, *P4* and *P5* region. **A** Structure of the *OsNRT2.2 *promoter. *P1,* *P2*, *P3*, *P4* and *P5* regions are indicated by red bars of different lengths, respectively. **B**, **C** Binding activity assay of OsCCA1 binding to *P2*, *P3*, *P4* and *P5* region via yeast one-hybrid system

To determine which region of the *OsNRT2.2* promoter sequence could be recognized and bound by OsCCA1, we fragmented the upstream sequence of the *OsNRT2.2* coding region into segments of 1000 bp (*P2*), 750 bp (*P3*), 500 bp (*P4*), and 250 bp (*P5*). These segments were subsequently cloned into the pAbai vector (Fig. 4A). For the binding test, each of these fragments was further analyzed using the Yeast One-Hybrid system. As shown in Fig. 4B, OsCCA1 could bind to *P2* and *P3* but not *P4* and *P5*. These results suggested that the OsCCA1 binding site may locate within the 500-1500 bp upstream sequence of the *OsNRT2.2* coding region.

**Fig. 5:**
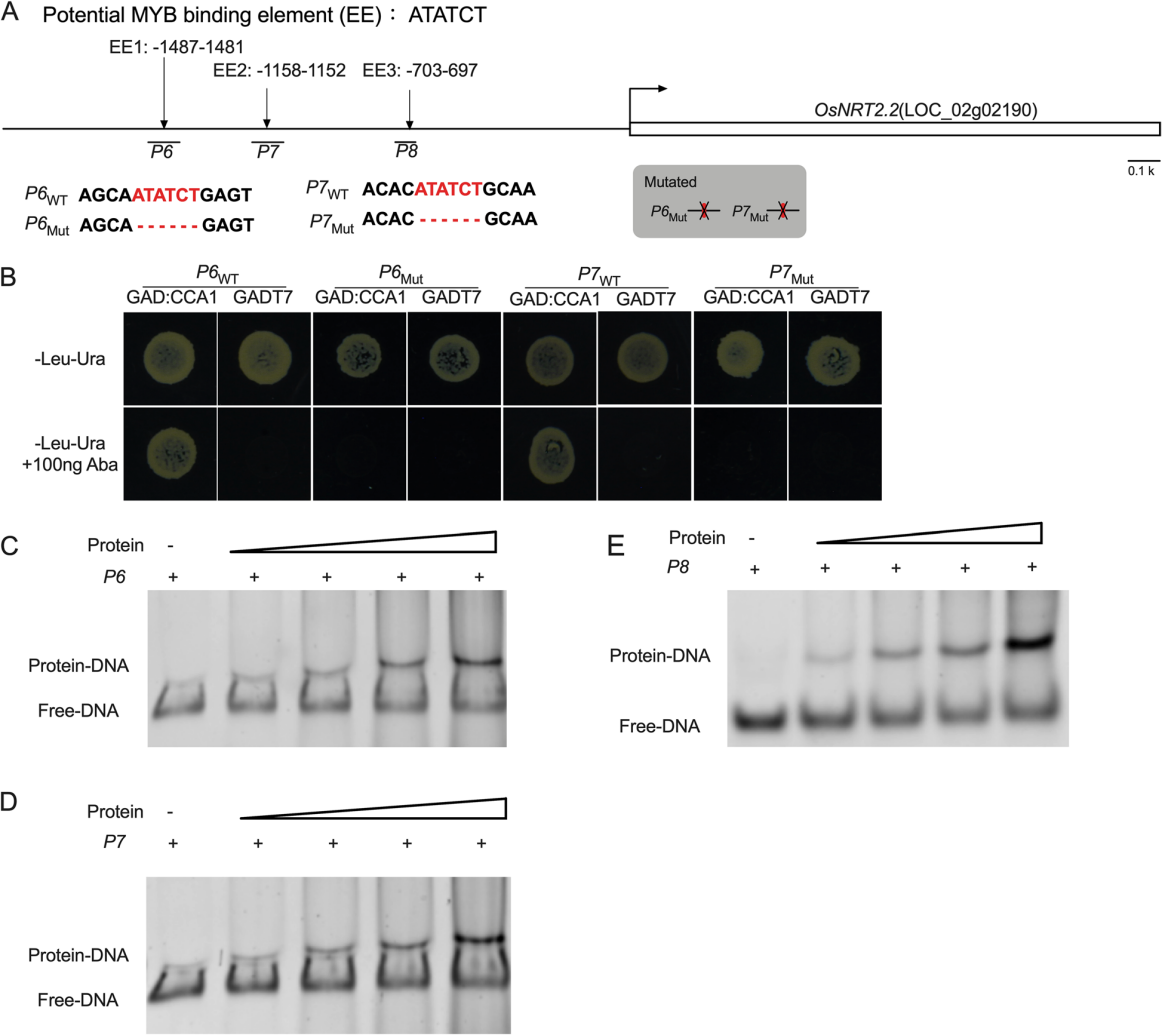
Binding activity analysis of OsCCA1 and potential MYB binding elements. **A** Potential MYB binding elements in the promoters of *OsNRT2.2*. Potential MYB-binding elements are noted in red, and the mutated base is indicated by a short horizontal line. **B** Binding activity analysis between OsCCA1 and *P6*_WT_, *P6*_Mut_, *P7*_WT_ and *P7*_Mut_ using yeast one-hybrid assays, respectively. **C**, **D**, **E**, Binding activity of OsCCA1 to *P6*, *P7* and *P8* region in EMSA system

In the 500-1500 bp upstream sequence of the *OsNRT2.2* coding region (Fig. 5A), three putative MYB-binding elements (EE) were identified, indicating that OsCCA1 could potentially bind to these elements on the *OsNRT2.2* promoter. Therefore, we subsequently investigated the other three fragments, *P6*, *P7* and *P8* in the *OsNRT2.2* promoter, which contain MYB-binding elements, using the Yeast One-Hybrid system and EMSA assay.

In the yeast one-hybrid assay, we observed that OsCCA1 could bind to *P6*_WT_ and *P7*_WT_ but not *P6*_Mut_ and *P7*_Mut_ (Mut: delete MYB-binding elements) (Fig. 5A and B). We did not assay *P8* in the yeast one-hybrid system due to self-activation. To further verify whether the EE in the *OsNRT2.2* promoter could be recognized by OsCCA1 using *P6, P7* and *P8*, we performed an EMSA assay using *P6*, *P7* and *P8*, respectively. As shown in Fig. 5C-E, all three fragments, *P6, P7* and *P8*, displayed mobility shifts when incubated with OsCCA1(Fig. S4). These results confirmed that OsCCA1 could directly bind to the EE in the *OsNRT2.2* promoter.

### Knockdown of OsCBL1 decreases the expression of OsCCA1 in rice plant

**Fig. 6:**
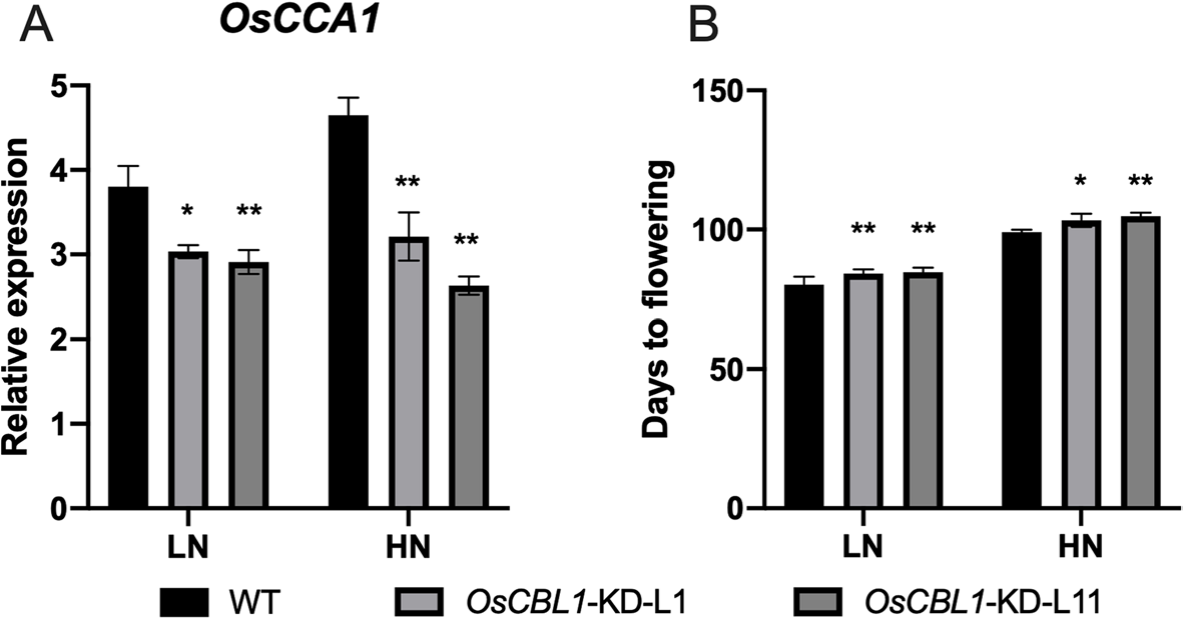
Knockdown of *OsCBL1* results in decreased expression of *OsCCA1 *and delayed flowering time. **A** The expression of *OsCCA1* in WT and *OsCBL1*-KD plants under HN and LN levels. *n* = 3 biologically independent samples. The error bars represent ± SDs. **p < 0.01 compared to the WT (t-test). **B** The flowering time of *OsCBL1*-KD and WT plants under HN and LN levels. *n* = 6 biologically independent samples. The error bars represent ± SDs. *p < 0.05, and **p < 0.01 compared to the WT (t-test)

The flowering time of *OsCBL1*-KD rice plants was delayed in comparison with WT rice plants under both HN and LN fields (Fig. 6B). This observation piqued our interest in exploring the relationship between *OsCBL1* and *OsCCA1*, given that *OsCCA1* is a vital component of the circadian clock in rice. We determined the transcript levels of *OsCCA1* in *OsCBL1*-KD and WT plants. As shown in Fig. 6A, knocking down *OsCBL1* resulted in a substantial decrease in the expression of OsCCA1 compared to WT plants. Considering the result that OsCCA1 restrained the expression of *OsNRT2.2* in the LUC assay (Fig. 3D), we speculated that *OsCBL1* may regulate the expression of *OsNRT2.2* by controlling *OsCCA1*.

### Knockdown of *OsCBL1* increases NUE under low nitrogen level

*OsCBL1-*KD and WT plants were cultivated in fields with high N (360 kg-N/ha) and low N (90 kg-N/ha) in fields, respectively. The WT plants exhibited better nitrogen- related agronomic traits under high N compared to low N (Fig. S5), indicating the successful application of nitrogen. Quantitative measurements of the traits related to NUE were completed with replicates. At the maturity stage, the plant height and effective panicle number in *OsCBL1-KD* plants were approximately the same as those in WT plants under LN level (Fig. S6). However, under the HN level, *OsCBL1*-KD rice plants showed significantly shorter plant height and fewer effective tiller numbers in comparison to WT (Fig. S6). Notably, the loss of effective tiller number in *OsCBL1*-KD plants from HN to LN was lower than that of WT plants, suggesting that *OsCBL1*-KD plants were less sensitive to the reduction of nutrient N in the environment. These results indicate that the knockdown of *OsCBL1* can improve the nitrogen tolerance of rice under LN conditions.

**Fig. 7:**
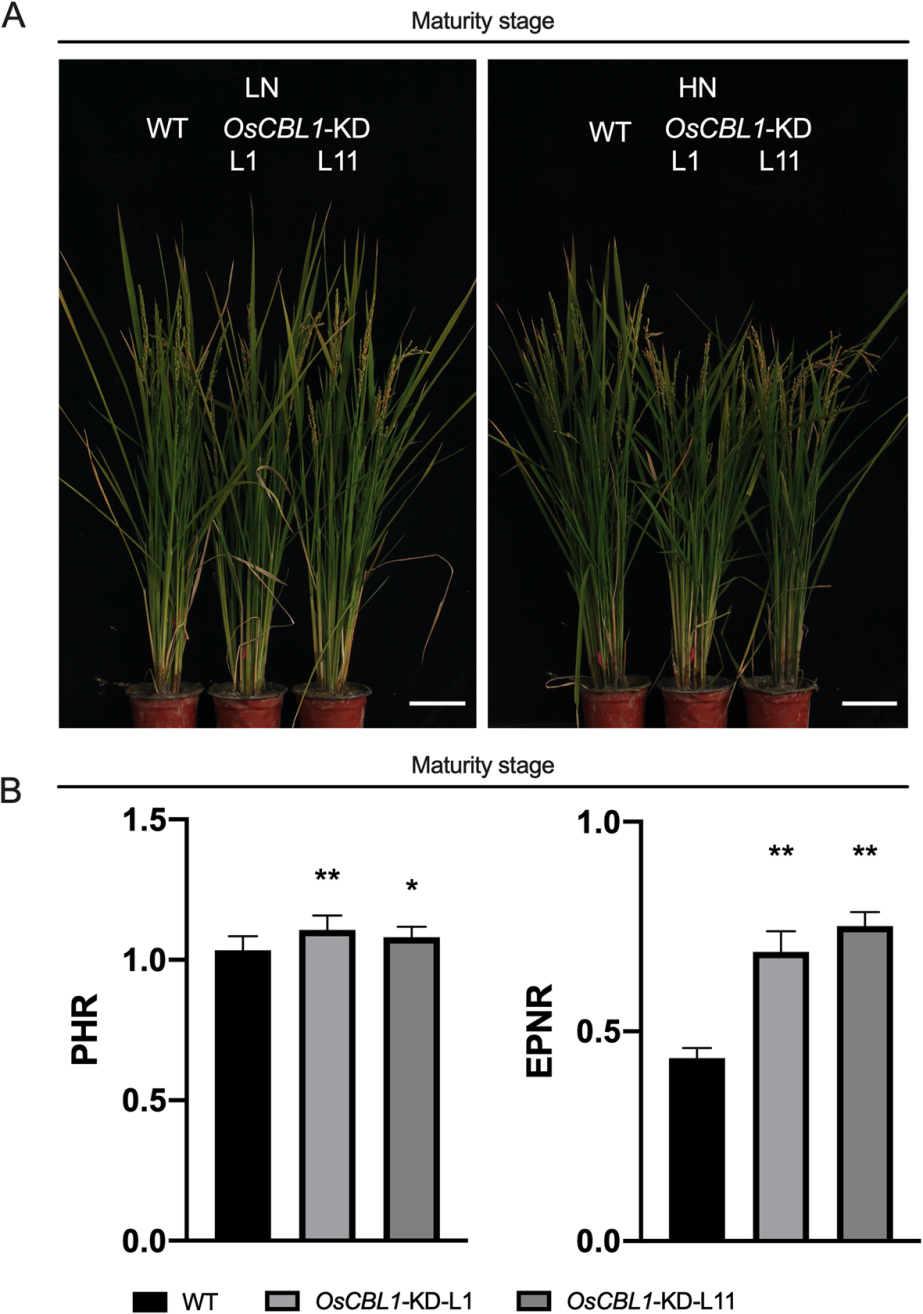
Growth phenotype of *OsCBL1*-KD and WT at maturity stage. **A** Growth phenotype of *OsCBL1*-KD and WT at maturity stage under HN (360 kg-N/ha) and LN (90 kg-N/ha) levels. Scale bars, 10 cm. **B** The plant height ratio (PHR) and effective panicle number ratio (EPNR) (LN/HN) of WT and *OsCBL1*-KD plants at the maturity stage. n ≥ 12 biologically independent samples. The error bars represent ± SDs. *p < 0.05, and **p < 0.01 compared to the WT (t-test)

**Fig. 8:**
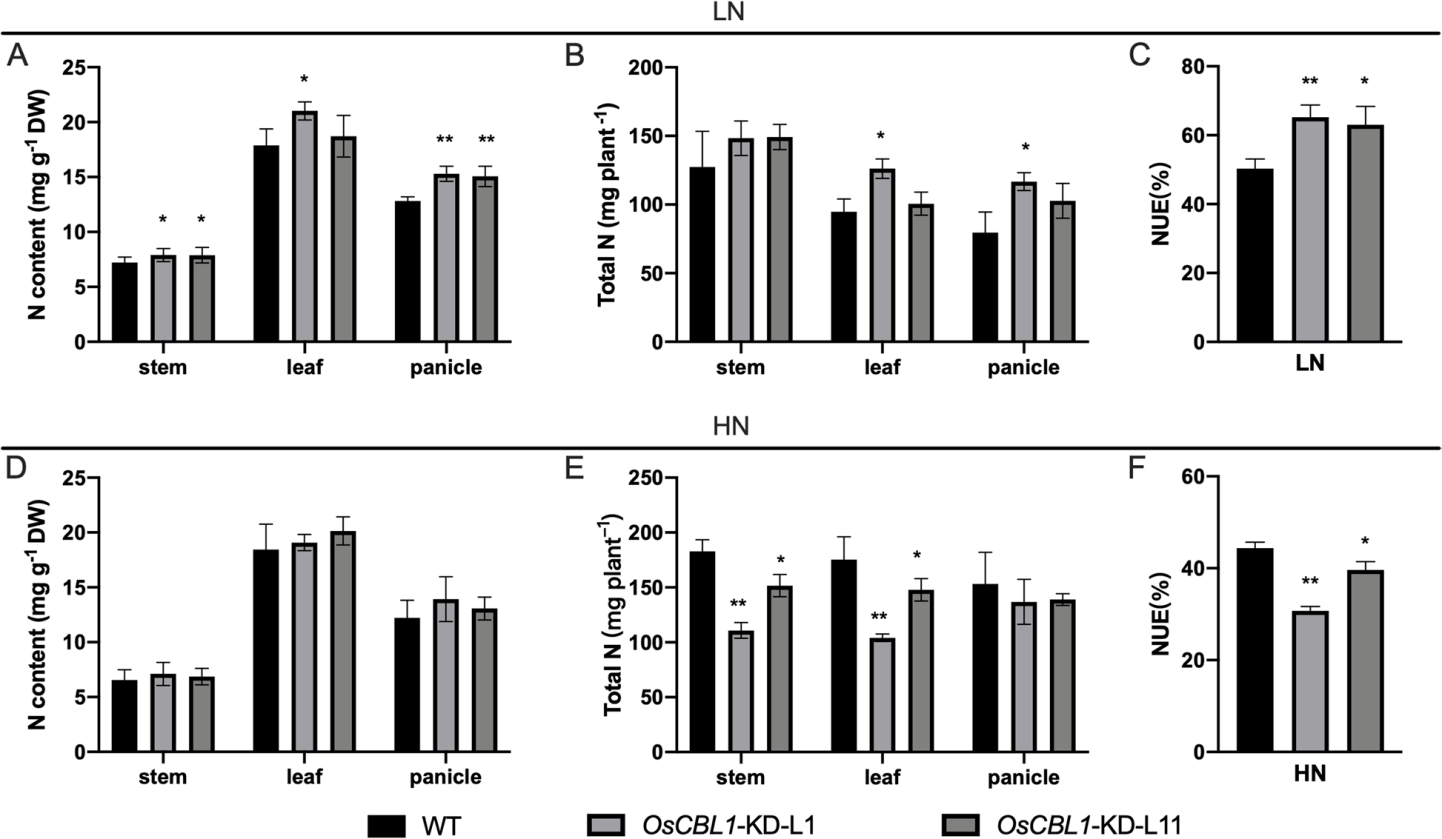
Knockdown of *OsCBL1* increases NUE under LN level. **A**, **B**, **D**, **E** The N content and total N of panicles, leaves and stems of WT and *OsCBL1*-KD plants at maturity stage under LN and HN levels. *n* ≥ 5 biologically independent samples. The error bars represent ± SDs. *p < 0.05, and **p < 0.01 compared to the WT (t-test). **C**, **F** The NUE of *OsCBL1*-KD and WT under LN (90 kg-N/ha) and HN (360 kg-N/ha) levels. *n* ≥ 5 biologically independent samples. The error bars represent ± SDs. *p < 0.05, and **p < 0.01 compared to the WT (t-test)

 Usually, rice NUE is evaluated by the plant height ratio (PHR) and effective panicle number ratio (EPNR) to assess rice NUE from LN to HN [18]. Therefore, we analyzed the PHR and EPNR of *OsCBL1*-KD and WT plants at the maturity stage. The *OsCBL1*-KD plants showed significantly higher PHR and EPNR (Fig. 7) than the WT, suggesting that *OsCBL1* plays a key role in regulating NUE. We further tested the nitrogen content and total nitrogen accumulation in panicles, leaves and stems at the maturity stage in both *OsCBL1-KD* plants and wild-type rice. As shown in Fig. 8, under the HN level, the N content in *OsCBL1*-KD plants was approximately the same as wild type, whereas the total nitrogen accumulation and NUE decreased due to the reduced biomass. However, under the LN level, the total N accumulation was higher in *OsCBL1*-KD plants than in WT plants, mainly due to higher N content rather than dry weight biomass (Fig. 8, Fig. S7). Additionally, under the LN condition, *OsCBL1-*KD plants increased NUE by 25.3-29.7% compared to wild type rice (Fig. 8C). Therefore, knocking down of *OsCBL1* could increase rice NUE under LN level but not HN level.

## Discussion

Besides being a nutrient element, nitrate is also discovered as an important signaling molecule that communicates between plants and the environment [23, 32]. In order to improve the NUE of crops, understanding the mechanisms and identifying the genes in plants that are involved in the process of sensing and responding to nitrogen availability is crucial [33]. Therefore, researchers continue to investigate the factors involved in nitrogen-related signal pathways, such as N uptake, assimilation, metabolism and regulation. Several nitrate transporter genes have been reported and identified in rice as effectors that could help increase rice grain yield and enhance NUE in the field. For example, *OsNRT1.1 A* has been found involved in regulating N utilization and flowering. Overexpression of *OsNRT1.1 A* greatly increases NUE and grain yield, and significantly shortens the maturation time [7]. Another nitrate-transporter gene in *indica* variety, *OsNRT1.1B*, can also improve the grain yield and NUE of *japonica* variety. In addition, OsNRT1.1B has been found interacting with OsSPX4 (phosphate response repressor), and synergistically activates nitrate and phosphate response genes to achieve N and P balance [8, 34]. Moreover, the nitrate transporter genes *OsNRT2.1/2.2* and *OsNRT2.3b* have also been reported to increase rice grain yield and NUE in the field recently [5, 6]. Though these nitrate transporter genes have been identified for the benefit of the NUE in rice, their upstream integrated regulatory mechanisms are rarely reported.

In this study, we found that the knocking down of *OsCBL1*, a calcium sensor gene, could promote the expression of *OsNRT2.2* (Fig. 2) under both HN and LN conditions. Given our previous findings that knocking down *OsCBL1* resulted in increased expression of *OsNRT2.2* under different nitrogen status [29], we believe that *OsCBL1* negatively regulates the expression of *OsNRT2.2.* However, OsCBL1 is known as a calcium sensor, belonging to Calcineurin B-like protein family, and has three EF-hand/calcium-binding motif [28]. Clearly, *OsCBL1* cannot directly regulate the expression of nitrate transporter *OsNRT2.2*. Therefore, this observation may provide a novel clue to discovering the upstream integrated regulatory mechanisms of nitrate transporter genes, and these results can also provide evidence for the s interplay between nitrate response and calcium-related signal pathways in rice.

**Fig. 9:**
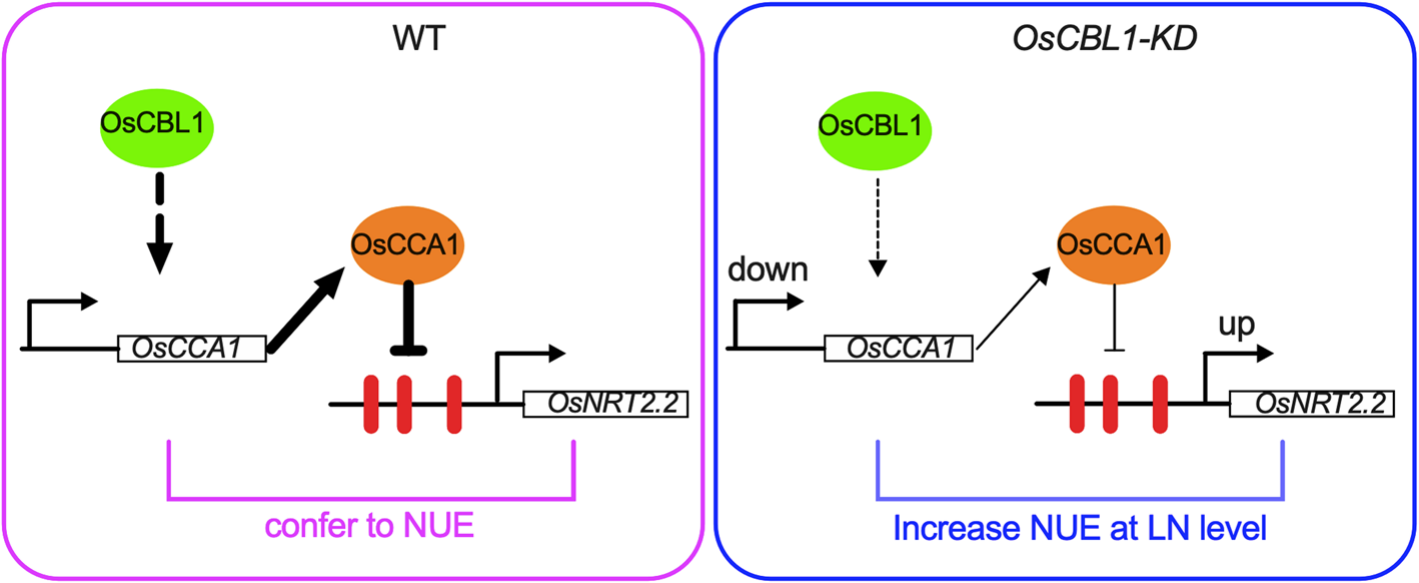
Knockdown of *OsCBL1* led to a decrease in *OsCCA1* expression, which increased both the transcript level of *OsNRT2.2* and N use efficiency (NUE) in rice. Potential MYB-binding elements in the *OsNRT2.2* promoter are marked with a red vertical line. The green solid circle indicates OsCBL1 protein. The orange solid circle indicates the OsCCA1 protein. Lines ending in arrowheads indicate positive regulation. Lines ending in blunted heads indicate negative regulation. The thickness of the lines indicates the controlling power of the downstream genes

 OsCCA1, a vital circadian rhythm component, could directly bind to the CCA1 binding sites (CBS) element in the *OsG1* promoter and precisely regulate the photoperiodic flowering of rice through the OsGI-Hd1 pathway [20]. In general, rice flowering time is promoted by low N and postponed by high N [1]. Wang et al. noted that mutation of *oscca1* could delay rice flowering time [31]. In addition, the regulation of heading date in rice by OsCCA1 is nitrogen-dependent. Therefore, OsCCA1 is also named as N-mediated heading date-1 (Nhd1). Recently, Zhang et al. found that the regulation of heading date in rice by OsCCA1/OsNhd1 is nitrogen-dependent, and dysfunction of *Nhd1* postponed rice flowering time in both HN and LN fields [21]. Li et al., on the other hand, found that *Nhd1* can regulate the root growth and NUE of rice by directly turning on the expression of *OsAMT1;3* and *OsNRT2.4* [22]. Here, for the first time we discovered that the circadian rhythm component OsCCA1 can directly bind to the *OsNRT2.2* promoter and influence the expression of *OsNRT2.2* as a negative effector in rice (Fig. 3). We also observed an improved NUE in the *OsCBL1*-KD plant with subsequent increased expression of *OsNRT2.2* (Figs. 2 and 8). This finding greatly enriched our understanding for OsCCA1-regulated pathway of NUE in rice. A previous study reported that LHY and CCA1 could bind to the CCA1 binding sites (AAAAATCT) and MYB-binding elements (ATATCT) in *Arabidopsis* [35, 36]. Previous studies have confirmed that OsCCA1 can regulate the expression of target genes by binding to CBS motifs [21, 31]; whereas, the binding of OsCCA1 to EE motifs for the regulation of target genes in rice has not yet been reported. In this study, we discovered that OsCCA1 can directly suppress the expression of *OsNRT2.2* by binding to EE motifs in the *OsNRT2.2* promoter (Figs. 3, 4 and 5). It has been reported in *Arabidopsis* that CCA1 can regulate the target genes by binding to CBS and EE motifs [35, 36]. Therefore, these results inclined that the binding motifs of OsCCA1 are conserved in rice and *Arabidopsis*. Taken together, our results showed that *OsCBL1*-KD results in a decreased expression of *OsCCA1.* As a negative regulator for *OsNRT2.2*, the limitation of OsCCA1 released the suppression over the target gene, and thereby elevated the transcript level of *OsNRT2.2* (Figs. 3, 6 and 9). However, the mechanism of how *OsCBL1* regulates the expression of *OsCCA1* remains a subject for further investigation.

Highly-yield of crop relies on high N application, while this is not sustainable in the long term considering the impacts on global environmental [33]. Reducing- N- fertilizer levels in fields and minimizing environmental pollution have become urgent objectives for sustainable development in the context of the Second Green Revolution. Thus, improving the NUE of crops under low N level is more meaningful and feasible for economic value and ecological benefits. It was previously reported that over-expression of *OsNRT2.1/2.2* can also increase rice NUE [5]. Additionally, the loss-of -function mutations in OsNhd1/OsCCA1 have been shown to prolong rice flowering time and increase N uptake and utilization efficiency [21]. The *nhd1* mutants exhibited the increased N accumulation and NUE in low-N supplement paddy fields via the delaying flowering time and prolonging growth period [21]. Here in *OsCBL1*-KD plants, the expression of *OsCCA1* was suppressed, and flowering time was delayed (Fig. 6), simultaneously, the expression of *OsNRT2.2* was enhanced (Fig. 2). These observations collectively these observations inspired us to explore the potential of NUE in *OsCBL1*-KD plants. For these *OsCBL1*-KD plants, significant improvements in both N accumulation and NUE at low N input were found in the current study (Fig. 8). These results are consistent with previous researches [21, 22], indicating that *OsCCA1* concomitantly regulates flowering time and NUE in rice.

Previous research found that *OsNRT2.1/2.2*-overexpression, driven by the native promoter (*pNAR2.1*), can increase the NUE of rice. However, when driven by the *pUbi* promoter, the NUE of rice was decreased by *OsNRT2.1/2.2*-overexpression. Additionally, it has been observed that *OsNRT2.1/2.2*-overexpression driven by its native promoter can increase NUE to a greater extent at the LN level than at the HN level [5]. These findings collectively suggested that controlling the fine-tuning of *OsNRT2.1/2.2* expression is essential for improving NUE in rice. In this study, the upregulation of expression of *OsNRT2.2* was governed by the down-regulated *OsCCA1* in *OsCBL1-KD* plants (Figs. 2, 3 and 6). Moreover, *OsCBL1-KD* plants also exhibited improved NUE at the LN level field (Fig. 9). This could be another effective pathway for regulating the fine expression of *OsNRT2.2* and, ultimately, improving the NUE of rice. However, despite the increased NUE under LN conditions, the grain yield was not increased in *OsCBL1-*KD plants. Our previous research reported that *OsCBL1* plays an important role in regulating plant growth, and *OsCBL1*-KD inhibited the growth of rice seedling [29]. In this study, slow plant growth was observed in *OsCBL1*-KD rice plants at the HN level (Fig. S6, Fig. 7), and increased NUE was found at the LN level primarily due to higher N content rather than dry weight biomass (Fig. 8, Fig. S7). The lack of increase in grain yield in *OsCBL1-*KD plants, despite the increase NUE under LN level may be attributed to the downregulation of *OsCBL1*, which disrupts the balance between plant growth and the surrounding nutrient environment. Consequently, the increased N accumulation in *OsCBL1*-KD rice does not result in an increment in biomass and grain yield under LN level field. In our experiments, the biomass ratio (LN/HN) of *OsCBL1*-KD plants is higher than that of WT plants under hydroponic conditions (Fig. 1C). Moreover, form HN to LN field, the loss of effective tiller number in *OsCBL1*-KD plants was less than that in WT (Fig. S6), revealing that *OsCBL1*-KD plants were more tolerant to LN treatment. This suggested that *OsCBL1* possesses the ability to efficiently utilize nutrients in poor soil. Further analysis will focus on the mechanism of how *OsCBL1* coordinates the balance between regulating plant growth and responding to the environmental nutrient impacts.

## Conclusion

In summary, our study revealed that reducing the expression of the *OsCBL1* in rice results in the increased expression of the nitrate high-affinity gene *OsNRT2.2*. This may be caused by the down-regulation of transcription factor *OsCCA1*, and ultimately leading to the NUE-enhancement under low nitrogen conditions. The current results reveal a possible interplay between nitrate response and calcium-related signal pathways in rice and suggest tha*t OsCBL1* is a promising candidate gene for crop nitrogen use efficiency under low nitrogen levels. The finding will contribute to the Sustainable Green Revolution in rice.

## Materials and methods

### Plant materials and growth conditions

The wild-type rice ShijinB and transgenic *OsCBL1*-knockdown (*OsCBL1*-KD) plants in this study were used as described before [29]. For hydroponic experiments, rice seeds of WT and *OsCBL1*-KD were sterilized with 5% (v/v) NaClO at room temperature for 30 min and then germinated in a dark incubator at 30 ℃ for 2–3 days after surface. Similar seedlings were transferred to an 8-L hydroponic box for an additional 7–30 days. The plants grew in a growth chamber with a photoperiod of 12 h (light)-12 h (dark) (~ 200µmolm^−2^ s^−1^) at 30 ℃/28 and 70% humidity. Kimura B nutrient solution is used as full nutrient solution at Ph 5.8 including macronutrients (in mM): (NH_4_)_2_SO_4_ (0.365), KH_2_PO_4_ (0.128), KNO_3_ (0.183), K_2_SO_4_ (0.086), Ca(NO_3_)_2_ (0.366), MgSO_4_·7H_2_O (0.548), Na_2_SiO_3_·9H_2_O (1.6) and micronutrients (in µM): MnCl_2_ · 4H_2_O (0.091), H_3_BO_3_ (46.2), (NH_4_) _6_Mo_7_O_24_ · 4H_2_O (0.145), ZnSO_4_ · 7H_2_O (0.77), CuSO_4_ · 5H_2_O (0.32) and Fe (II) -EDTA (40). To create different nitrogen concentrations in the solution, Ca(NO_3_)_2_ was replaced by CaCl_2_ and the final concentrations of KNO_3_ in HN and LN conditions were adjusted to 0.2 mM and 5 mM, respectively. The K^+^ concentration was balanced with KCl to maintain consistency among different conditions. The HN and LN solutions were renewed every two days.

For field experiments, plants were planted at two different places and seasons specifically for transgenic rice: the one place is the Nanchang University experimental field in Jiangxi Province, where rice plants grew from April to August in 2022. The other place is Jiangxi Academy of Agricultural Sciences experimental field in Jiangxi Province and rice plants were grown from June to October in 2022. Rice seeds were sown on seedbeds and grown for 30 days, then seedlings were transplanted to the HN (360-kg/ha) and LN (90-kg/ha) fields until the harvest stage. Each variety was planted 10 plants × 5 rows at a spacing of 20 cm under low and high nitrogen conditions. The nitrogen fertilizer (urea) was applied one day before transplanting, during tillering, and at the flowering stage with 50%, 25% and 25% of total N, respectively. Biomass and N values obtained at three repeated points in each plot were used to calculate dry weight and N content. Three random plots were designed for each line in this experiment.

### Dry weight, N accumulation and calculation of NUE

For biomass and total nitrogen content, WT and *OsCBL1*-KD plants were harvested at the maturity stage from HN and LN fields, respectively. Each individual plant of WT and *OsCBL1*-KD was separated into leaves, stems and panicles. These tissues were dried in an oven first at 105 ℃ for 30 min and sat at 85 ℃ until reaching a constant weight. After drying, the tissues were ground into powder using a crusher. 0.2 g of rice tissue powder was dissolved with 5 ml of sulfuric acid for 4–5 h at 350 ℃. The total N concentration of tissues was analyzed by indophenol blue method and determined by auto discrete analyzers (SmartChem200, westco, France) [37]. Total dry biomass and nitrogen accumulation were calculated as the sum of the biomass and nitrogen accumulation of the three plant parts, respectively. The NUE was analyzed following the same method as previously described [21]. Nitrogen use efficiency was calculated as the following equation:

$$\mathrm{Nitrogen}\;\mathrm{Use}\;\mathrm{Efficiency}\;\left(\mathrm{NUE},\;\%\right)=\frac{\mathrm{total}\;\mathrm{acquired}\;\mathrm N\;\mathrm{per}\;\mathrm{Plant}\;\times\mathrm{number}\;\mathrm{of}\;\mathrm{plants}\;\mathrm{per}\;\mathrm{ha}}{\mathrm{total}\;\mathrm{amount}\;\mathrm{of}\;\mathrm{applied}\;\mathrm{fertilizer}\;\mathrm N\;\mathrm{per}\;\mathrm{ha}}\times100\%$$

### RNA isolation and qPCR analysis

Total RNA isolation and RT-qPCR analysis were completed as previously described [29]. Briefly, total RNA was isolated using TRNzol Universal (TIANGEN, Cat no. DP424). Reverse transcription reactions were performed using FastKing RT Kit (TIANGEN, Cat no. KR116). The qPCR assay was performed under StepOnePlus Real-Time PCR system with Power SYBR Green Master Mix (Applied Biosystems). The qPCR data on qPCR was analyzed by Graph Pad Prism 8. For every gene, each data point was obtained on biological sample triplicate. The relative expression of target genes was normalized by the housekeeper gene Actin1. The related primers are listed in Table S2.

### Yeast-one hybrid assay

For library screening assay, promoter of *OsNRT2.2* (1.5 kb upstream regions from the start codon) was amplified and cloned into pAbai fused with AurR gene as a reporter. The constructs were linearized at the *XhoI* or *AflII* sites and integrated into the chromosome of bait yeast strain AH109. The rice nuclear library was constructed by OE Biotech (Shanghai) by cloning the open reading frames of rice nuclear genes into the pGADT7 vector, which fused the open reading frames with the GAL4 activation domain. The resulting constructs were transformed into Y187 yeast cells. The bait strain AH109 and the prey strain Y187 were mated and screened using the selective medium SD/-Leu/-Ura/Aba (Aureobasidin A, 80–120 ng). Furthermore, an additional assay was performed using the Matchmaker Gold Yeast One-Hybrid Library screening System (Clontech, Takara).

For Y1H assay, the full-length CDS of *OsCCA1* was cloned into the pGADT7 vector to express fusion proteins containing the yeast GAL4 transcription activation domain. The 1500 bp (*P1*), 1000 bp (*P2*), 750 bp (*P3*), 500 bp (*P4*) and 250 bp (*P5*) upstream sequence of the *OsNRT2.2* coding region, as well as the *P6* (100 bp DNA fragment containing MYB binding element), *P6*_mut_ (delete the MYB binding element in *P6*), *P7* (100 bp DNA fragment containing MYB binding element), *P7*_mut_ (delete the MYB binding element in *P7*), *P8* (64 bp DNA fragment containing MYB binding element) regions in the *OsNRT2.2* promoter were cloned into the pAbai vector, respectively. The Y1H Gold yeast strain was used as receptor. The further assay was performed using the Matchmaker Gold yeast One-Hybrid Library screening System (Clontech, Takara). The related primers are listed in Table S2.

### Luciferase activity assay in rice protoplasts

The isolation and transformation of rice protoplasts were performed according to previously published methods [38]. Rice shoots aged 10–15 days were used to extract protoplasts. The shoot pieces were digested in enzymatic digestion solution for 4 h at 28 °C. The protoplasts were then washed twice with W5 solution (154 mM NaCl, 125 mM CaCl_2_, 5 mM KCl, 0.18 mM KH_2_PO_4_ and 2 mM MES at pH5.7) and placed on ice for 30 min. Protoplasts were resuspended with MMG solution (0.4 M mannitol, 15 mM MgCl_2_ and 4 mM MES at pH 5.7) and used for transfection. The transfection solution (volume ratio, plasmids: protoplasts : PEG4000 solution = 1 : 10 : 11) was incubated at room temperature for 30 min in the dark. Then, 500 ul of W5 solution was added to terminate the reaction.

The full length CDS of *OsCCA1* was amplified from cDNA of WT and cloned into *pCAMBIA1301* vector for generating the effector. The 1500 bp upstream sequence of *OsNRT2.2* coding region was fused with firefly luciferase (*LUC*) sequences and introduced to the *pGreen-II0800* vector to generate the reporter. Then, the *pCAMBIA1301* carried with effector and *pGreen-II0800* carried with reporter were co-transfected into rice protoplasts. The *pCAMBIA1301* vector without *OsCCA1* was taken as the negative control. The Renilla luciferase (REN) was taken as a reference. The protoplast protein was extracted and used for the detection of REN and LUC activities by Dual-Luciferase Reporter Assay System (Promega, E1910) after being incubated in W5 solution for 16–20 h at room temperature in the dark. All the related primers are listed in Table S2.

### EMSA assay

Full length CDS of *OsCCA1* was cloned into the *pET-28a* vector and transformed into BL21 strain of *Escherichia coli* to express His-CCA1 fusion protein. The expression and purification of recombinant protein were performed using Ni-NTA 6FF (Sangon Biotech). The promoter sequence of the *OsNRT2.2* was cloned and purified from Wild-type rice DNA. The EMSA assays were performed as described previously [39]. *P6* (100 bp DNA fragment containing MYB binding element), *P7* (100 bp DNA fragment containing MYB binding element) and *P8* (64 bp DNA fragment containing MYB binding element) were incubated with His-OsCCA1 protein in binding buffer (10 mM Tris–HCl [pH 7.5], 50 mM KCl, 1 mM DTT) at 4℃ for 40 min, respectively. 4% TBE-polyacrylamide gels were used for electrophoretic assay. Electrophoresis was performed in TBE buffer at 120 V for 60 min at 4 ℃. Gels were stained with ethidium bromide (0.5 mg/mL) and photographed (Fig. S5, Fig. S6). The related primers are listed in Table S2.

### Data analysis

Experimental data were collected for calculating averages and standard deviation (SD), and the number of biological replicates is indicated in the legend of each figure. Statistical significance between the transgenic lines and WT plants was determined by Student’s t-test at P ≤ 0.05. All statistical analysis was performed using Prism8 statistical software.

### Supplementary Information

**Additional file 1: Table S1.** Putative transcription factor for *OsNRT2.2 *by Y1H Library Screening. **Table S2.** Primers used in this study. **Fig. S1.** The expression of *OsNRTs*in WT and *OsCBL1*-KD plants. Quantitative PCR analysis of the expression of *OsNRT1.1A, OsNRT1.2, OsNRT1.4, OsNRT1.5A,OsNRT1.7, OsNRT2.1, OsNRT2.3, OsNRT2.4 *and *OsNRT2.5 *in roots. *n* = 3 biologically independent samples. The error bars represent ± SDs. *p < 0.05, and **p < 0.01 compared to the WT (t test). **Fig. S2.** The conserved structural domain analysis of CCA1 amino acid sequence analyzed by SMART and SWISSMODEL. Background black bar denotes the MYB binding domain. **Fig. S3.** The original and uncropped gel image of Fig 3B. **Fig. S4.** The original and uncropped gel image of Fig 5C (A), Fig 5D (B), Fig 5E (C). **Fig. S5.** The plant height and effective pancile number of WT and *OsCBL1*-KD plants at maturity stage under HN and LN levels. n ≥ 12 biologically independent samples. The error bars represent ± SDs. *p < 0.05, and **p < 0.01 compared to the WT (t test). **Fig. S6.** The biomass of WT and *OsCBL1*-KD plants at maturity stage under HN and LN levels. n ≥ 4 biologically independent samples. The error bars represent ± SDs. *p < 0.05, and **p < 0.01 compared to the WT (t test). **Fig. S7.** The biomass of WT and *OsCBL1*-KD plants at maturity stage under HN and LN levels. n ≥ 4 biologically independent samples. The error bars represent ± SDs. *p < 0.05, and **p < 0.01 compared to the WT (t test).

## Data Availability

Sequence of *OsCBL1*, *OsCCA1*, *OsNRT2.2*, *OsNRT1.1 A*, *OsNRT1.2*, *OsNRT1.4*, *OsNRT1.5 A*, *OsNRT1.7*, *OsNRT2.1*, *OsNRT2.3*, *OsNRT2.4* and *Actin1*were deposited in Rice Genome Annotation Project (rice.uga.edu). These genes accession numbers are Os10g0564800 (*OsCBL1*), Os08g0157600 (*OsCCA1*), Os02g0112600 (*OsNRT2.2*), Os03g023590(*OsNRT1.1 A*), Os03g0235900 (*OsNRT1.2*), Os01g0556700 (*OsNRT1.4*), Os02g0689900 (*OsNRT1.5 A*), Os01g0913300(*OsRT1.7*), Os02g0112100 (*OsNRT2.1*), Os01g0704100 (*OsNRT2.3*), Os01g0547600 (*OsNRT2.4*), Os03g0718100 (*Actin1*).

## Abbreviations

NUE: Nitrogen use efficiency
LN: Low nitrogen
OsCBL1: Calcineurin B-like protein-1
*OsCBL1-*KD: *OsCBL1*-knockdown
N: Nitrogen
NO_3_^−^: Nitrate
HN: High nitrogen
MYB: Myeloblastosis
EE: MYB-binding elements
