# Supplementary material for: OsCBL1 modulates rice nitrogen use efficiency via negative regulation of OsNRT2.2 by OsCCA1
Source: BMC Plant Biol. 2023 Oct 18;23:502. doi: 10.1186/s12870-023-04520-4 (PMC10583366; doi:10.1186/s12870-023-04520-4)
Supplement: Supplementary file 1 — Additional file 1: Table S1. Putative transcription factor for OsNRT2.2 by Y1H Library Screening. Table S2. Primers used in this study. Fig. S1. The expression of OsNRTsin WT and OsCBL1-KD plants. Quantitative PCR analysis of the expression of OsNRT1.1A, OsNRT1.2, OsNRT1.4, OsNRT1.5A,OsNRT1.7, OsNRT2.1, OsNRT2.3, OsNRT2.4 and OsNRT2.5 in roots. n = 3 biologically independent samples. The error bars represent ± SDs. *p < 0.05, and **p < 0.01 compared to the WT (t test). Fig. S2. The conserved structural domain analysis of CCA1 amino acid sequence analyzed by SMART and SWISSMODEL. Background black bar denotes the MYB binding domain. Fig. S3. The original and uncropped gel image of Fig 3B. Fig. S4. The original and uncropped gel image of Fig 5C (A), Fig 5D (B), Fig 5E (C). Fig. S5. The plant height and effective pancile number of WT and OsCBL1-KD plants at maturity stage under HN and LN levels. n ≥ 12 biologically independent samples. The error bars represent ± SDs. *p < 0.05, and **p < 0.01 compared to the WT (t test). Fig. S6. The biomass of WT and OsCBL1-KD plants at maturity stage under HN and LN levels. n ≥ 4 biologically independent samples. The error bars represent ± SDs. *p < 0.05, and **p < 0.01 compared to the WT (t test). Fig. S7. The biomass of WT and OsCBL1-KD plants at maturity stage under HN and LN levels. n ≥ 4 biologically independent samples. The error bars represent ± SDs. *p < 0.05, and **p < 0.01 compared to the WT (t test). [file 12870_2023_4520_MOESM1_ESM.pdf]

OsCBL1 modulates rice nitrogen use efficiency via negative regulation of *OsNRT2.2* by OsCCA1

Supplementary tables

Table S1: Putative transcription factor for *OsNRT2.2* by Y1H Library Screening

Gene ID	Commentary
Os01g0678600	Ribosomal protein S20 family protein
Os06g0665500	Peptidase M20 domain containing protein.
Os08g0157600	MYB transcription factor, Circadian clock
Os11g0171300	Fructose-bisphosphate aldolase, chloroplast precursor
Os02g0805600	Similar to Alcohol dehydrogenase, zinc-containing.
Os01g0839900	Thaumatin, pathogenesis-related family protein.
Os10g0555700	Beta-expansin
Os11g0707100	Hypothetical conserved gene.
Os08g0487800	Similar to Heat-shock protein precursor
Os03g0285700	Cytosolic ascorbate peroxidase, Salt tolerance
Os06g0219900	Similar to Phi-1 protein.
Os05g0207400	Zinc finger, RING/FYVE/PHD-type domain containing protein.
Os01g0844400	Zinc finger, DHHC-type domain containing protein.
Os11g0133500	S-Domain kinase-2
Os10g0535600	Similar to Hydrolase.

Os01g0256500	Similar to ZnI
Os02g0264300	Conserved hypothetical protein.
Os12g0239200	Conserved hypothetical protein.

Table S2: Primers used in this study.

Primers for qPCR	
Primer name	sequence (5’-3’)
qNRT2.1-F	AATTCTGCGACCGAGACCAG
qNRT2.1-R	CCGTTTGCAACAAGGACGTG
qNRT2.2-F	GCATGCATGTATAAACTGTTGAACG
qNRT2.2-R	AGGCCTTTCTCCATGACGAC
qNRT1.2-F	TTGGTGGTCTTGAGGATG
qNRT1.2-R	GCTTAATCGCTGCTTCTT
qNRT1.4-F	ACCAGTAGTCATTGGCTAT
qNRT1.4-R	ATGATAACCTGGAGTGAACA
qNRT1.5A-F	AATAAGAGGTCGCCTACAG
qNRT1,5A-R	TGAGTGGTCAGTAACAGTC
qNRT1.7-F	ATGGAGTGTGAGTGATAGC
qNRT1.7-R	GTATGACCTGTGCTTACTTG
qNRT2.5-F	GGGCACTATACGCTAGTAC
qNRT2.5-R	GAGCAAACCACCAACAAG
qNRT2.4-F	AAAGGTCGCTGGGCGTGGTG

qNRT2.4-R	CCTGGACCCGCTGAAGAAGAG
qNRT1.1A-F	CCCACACCAAGCAATTCAGG
qNRT1.1A-R	GTCTTCACCTCCTCCACGTC
qNRT2.3-F	GCCATCCACAAGATCGGTAG
qNRT2.3-R	TGTGGAGCTTCCCGTAGTTG
qCCA1-F	GGGTCGTCTGGCTTTTGAT
qCCA1-R	CGGTACCCTGTTCTCCTTC
qACTIN1-F	ACCATTGGTGCTGAGCGTTT
qACTIN1-R	CGCAGCTTCCATTCCTATGAA

Primers for Yeast-one-hybrid assays

Primer name	sequence (5'-3')
pAbai-OsNRT2.2-p1-SacI-F	gaaaagcttgaattcgagctcGCCAGATTTAGCAATATCTGA GTCC
pAbai-OsNRT2.2-p1-SacI-R	agatccccgggtaccgagctcGCTTTGGATCGCGCATCG
pAbai-OsNRT2.2-p2-SacI-F	gaaaagcttgaattcgagctcAAAGTCGGCCGAAGAATTGC
pAbai-OsNRT2.2-p2-SacI-R	agatccccgggtaccgagctcGCTTTGGATCGCGCATCG
pAbai-OsNRT2.2-p3-SacI-F	gaaaagcttgaattcgagctcGACACGCCGTTGCTTTGC
pAbai-OsNRT2.2-p3-SacI-R	agatccccgggtaccgagctcGCTTTGGATCGCGCATCG
pAbai-OsNRT2.2-p4-SacI-F	gaaaagcttgaattcgagctcTTCTGTGGACAGATACAGAAC ATAACA
pAbai-OsNRT2.2-p4-SacI-R	agatccccgggtaccgagctcGCTTTGGATCGCGCATCG
pAbai-OsNRT2.2-p5-SacI-F	gaaaagcttgaattcgagctcAGAGATCAGAGTCGAGACTGT

	CAACG
pAbai-OsNRT2.2-p5-SacI-R	agatccccgggtaccgagctcGCTTTGGATCGCGCATCG
pAbai-OsNRT2.2-p6-SacI-F	gaaaagcttgaattcgagctcTAAAAAATACCTGCAGGTGCTCTAGT
pAbai-OsNRT2.2-p6-SacI-R	agatccccgggtaccgagctcATATAGCTTCGGTTGAAGTTTGTTAGTT
pAbai-OsNRT2.2-p7-SacI-F	gaaaagcttgaattcgagctcATATGATGTGTATGGGAATTGGGA
pAbai-OsNRT2.2-p7-SacI-R	agatccccgggtaccgagctcGAGAGGCACATTTTTCCATTAAA

Primers for transient expression assay

Primer name	sequence (5'-3')
pGreenII0800-OsNRT2.2-p1-F	ttcctgcagcccgggggatccGCCAGATTTAGCAATATCTGAGTCC
pGreenII0800-OsNRT2.2-p1-R	cgctctagaactagtggatccGCTTTGGATCGCGCATCG
pCAMBIA1301-OsCCA1-F	caggtcgactctagaggatccATGGAGATTAATTCCTCTGGTGAGG
pCAMBIA1301-OsCCA1-R	gatctgcaggtcgacggatccTCATGTCGATGCTTCGCTCTC

Primers for EMSA

Primer name	sequence (5'-3')
pET28a-OsCCA1-BamHI-F	cagcaaatgggtcgcggatccATGGAGATTAATTCCTCTGGT

	GAGG
pET28a-OsCCA1-BamHI-R	acggagctcgaattcggatccTGTCGATGCTTCGCTCTCAA
p6	TAAAAAATACCTGCAGGTGCTCTAGTTCAACTGA
	AGGGCCAGATTTAGCAATATCTGAGTCCTTCAAA
	TAAATTCATCAACTAACAAACTTCAACCGAAG
p7	ATATGATGTGTATGGGAATTGGGATGCTGAAAAC
	AAATGCAACTGGACACATATCTGCAACTTGTAAA
	TAACTTTTATTTCTTTTAATGGAAAAAATGTG
p8	CTTCAGGCCGTCATGAAAAAAAACCAGTGCATAT
	CTCATGGCAAGAAGAAGTGCAGGTTGTGAA

Legends of Supplementary Figures

Fig. S1. The expression of *OsNRTs* in WT and *OsCBL1*-KD plants. Quantitative PCR analysis of the expression of *OsNRT1.1A, OsNRT1.2, OsNRT1.4, OsNRT1.5A, OsNRT1.7, OsNRT2.1, OsNRT2.3, OsNRT2.4* and *OsNRT2.5* in roots. n = 3 biologically independent samples. The error bars represent ± SDs. $*p < 0.05$, and $**p < 0.01$ compared to the WT (t test).

Fig. S2. The conserved structural domain analysis of CCA1 amino acid sequence analyzed by SMART and SWISSMODEL. Background black bar denotes the MYB binding domain.

Fig. S3 The original and uncropped gel image of Fig 3B.

Fig. S4 The original and uncropped gel image of Fig 5C (A), Fig 5D (B), Fig 5E (C).

Fig. S5. The plant height and effective pancile number of WT and *OsCBL1*-KD plants at maturity stage under HN and LN levels. n ≥ 12 biologically independent samples. The error bars represent ± SDs. *p < 0.05, and **p < 0.01 compared to the WT (t test).

Fig. S6. The biomass of WT and *OsCBL1*-KD plants at maturity stage under HN and LN levels. n ≥ 4 biologically independent samples. The error bars represent ± SDs. *p < 0.05, and **p < 0.01 compared to the WT (t test).

Fig. S7 The biomass of WT and *OsCBL1*-KD plants at maturity stage under HN and LN levels. n ≥ 4 biologically independent samples. The error bars represent ± SDs. *p < 0.05, and **p < 0.01 compared to the WT (t test).

Supplementary Figures

Fig. S1

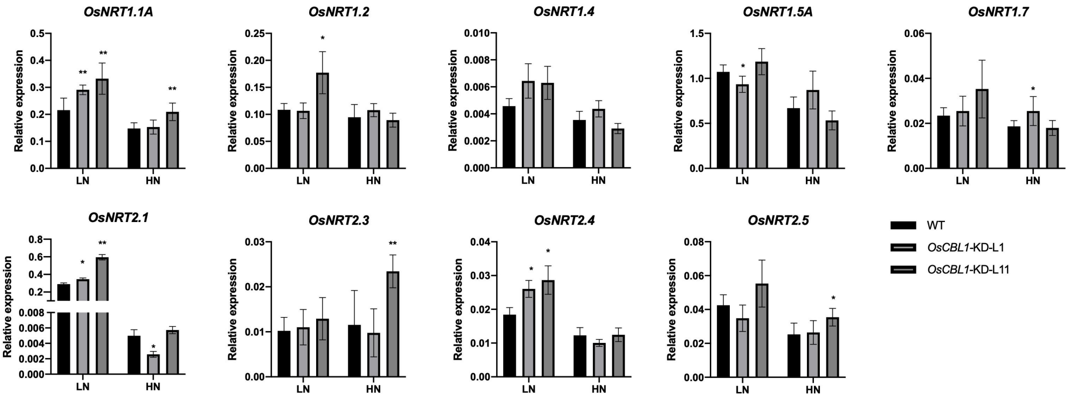

Fig. S1 The expression of *OsNRTs* in WT and *OsCBL1*-KD plants. Quantitative PCR analysis of the expression of *OsNRT1.1A, OsNRT1.2, OsNRT1.4, OsNRT1.5A, OsNRT1.7, OsNRT2.1, OsNRT2.3, OsNRT2.4* and *OsNRT2.5* in roots. n = 3 biologically independent samples. The error bars represent ± SDs. *p < 0.05, and **p < 0.01 compared to the WT (t test).

Fig. S2

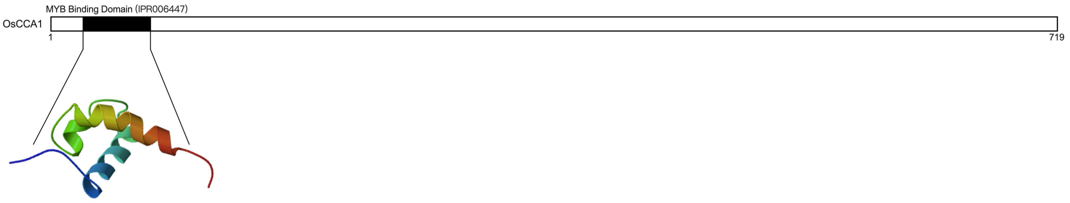

Fig. S2 The conserved structural domain analysis of CCA1 amino acid sequence analyzed by SMART and SWISSMODEL. Background black bar denotes the MYB binding domain.

Fig. S3

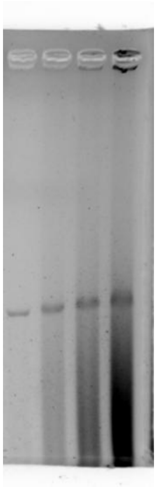

Fig. S3 The original and uncropped gel image of Fig 3B.

Fig. S4

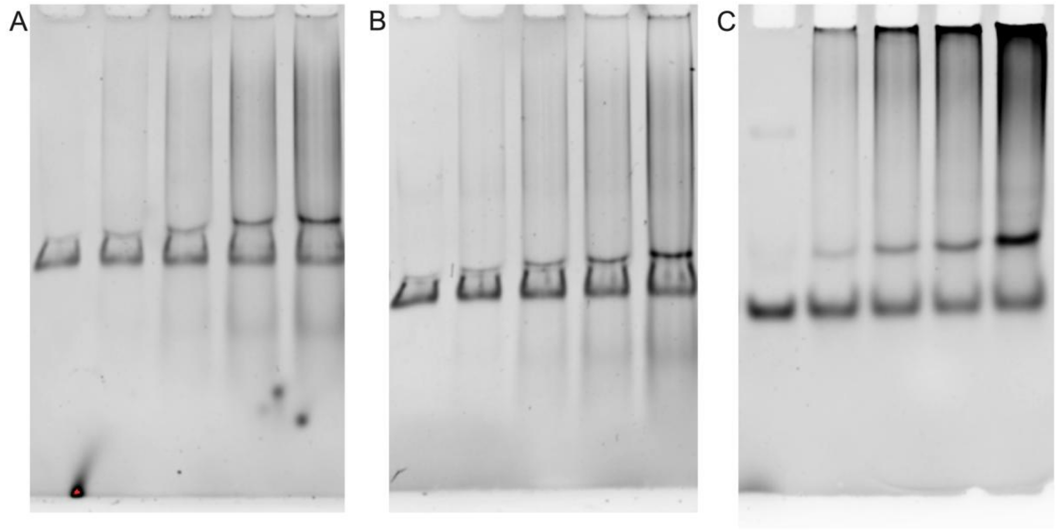

Fig.S4 The original and uncropped gel image of Fig 5C (A), Fig 5D (B), Fig 5E (C).

Fig. S5

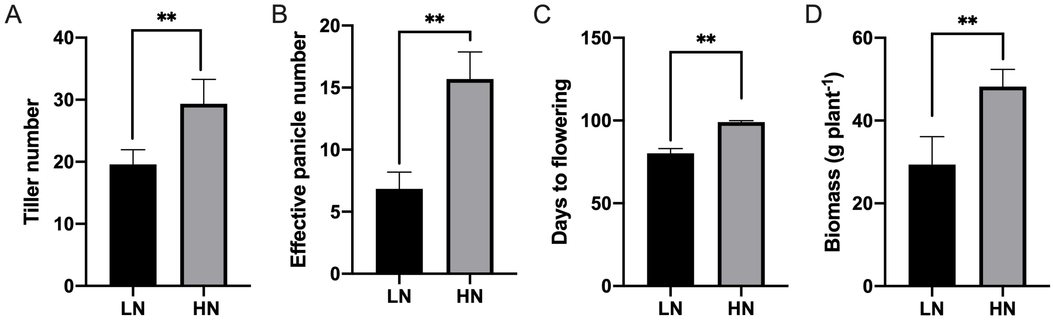

Fig. S5 Phenotypes of WT plants at maturity under HN and LN levels. The tiller number (A), effective panicle number (B), flowering time (C) and biomass (D) of WT plants at maturity under HN and LN levels. $n \geq 4$ biologically independent samples. The error bars represent ± SDs. **$p < 0.01$ compared to the LN (t test).

Fig. S6

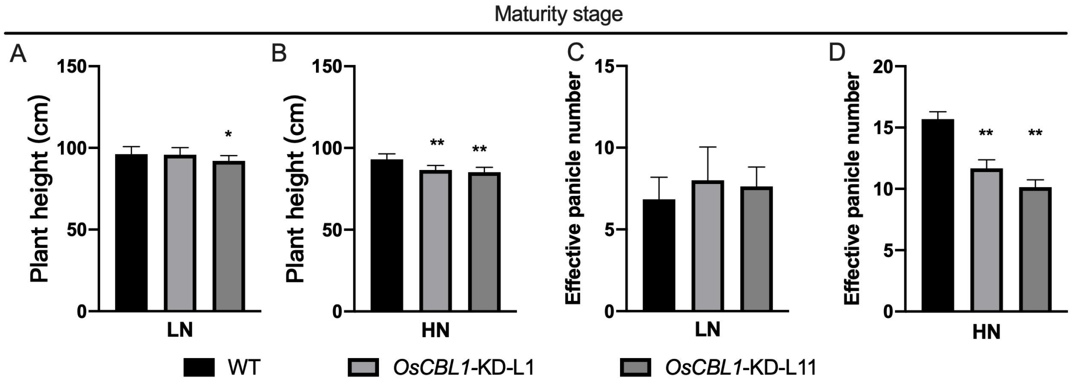

Fig. S6 The plant height and effective pancile number of WT and *OsCBL1*-KD plants at maturity stage under HN and LN levels. n ≥ 12 biologically independent samples. The error bars represent ± SDs. *p < 0.05, and **p < 0.01 compared to the WT (t test).

Fig. S7

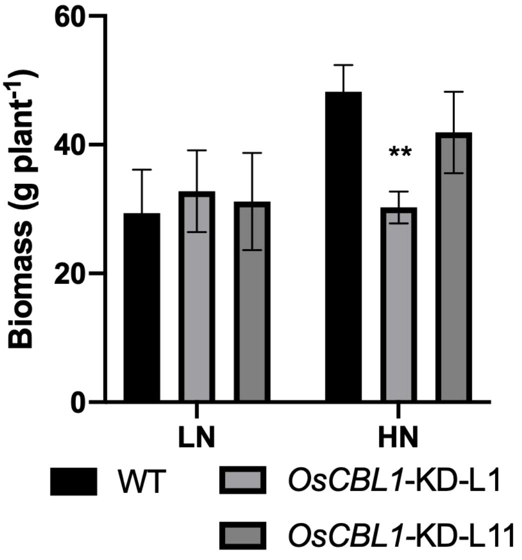

Fig. S7 The biomass of WT and *OsCBL1*-KD plants at maturity stage under HN and LN levels. n ≥ 4 biologically independent samples. The error bars represent ± SDs. *p < 0.05, and **p < 0.01 compared to the WT (t test).
